# Nonlinear propagation of ultrasounds in bubbly viscoelastic media: A study on the influence of the medium properties on the nonlinear parameter^☆^

**DOI:** 10.1016/j.ultsonch.2025.107603

**Published:** 2025-10-07

**Authors:** Elena V. Carreras-Casanova, María Teresa Tejedor-Sastre, Christian Vanhille

**Affiliations:** NANLA Research Group, Universidad Rey Juan Carlos, Calle Tulipán, s/n, Móstoles, 28933, Madrid, Spain

**Keywords:** Nonlinear acoustics, Bubbly viscoelastic media, Nonlinear parameter, Numerical simulations, Soft tissues, Ultrasound

## Abstract

A mathematical model that describes nonlinear ultrasonic wave propagation in bubbly viscoelastic media is developed by coupling the acoustic wave equation with a modified Rayleigh–Plesset equation, formulated in terms of bubble volume variation and incorporating the linear Kelvin–Voigt viscoelastic model. This formulation enables direct analysis of the influence of viscoelastic properties under soft tissue conditions. The differential system is numerically solved to investigate the transition from linear to nonlinear regimes in representative viscoelastic fluids and media that vary in shear elasticity. Laws of harmonic amplitudes vs. excitation at the source are defined from their polynomial fits. They confirm this nonlinear trend and reveal the influence of elasticity. To quantify these effects, the nonlinear parameter β is computed using a finite amplitude method. Our results show that increasing shear elasticity significantly attenuates nonlinear propagation and suppresses harmonic generation, with β decreasing as the shear modulus increases. In contrast, viscosity exhibits only a minor influence within the range studied in this work. These findings demonstrate that β is highly sensitive to the mechanical properties of the medium and can serve as an effective indicator to characterize the nonlinear acoustic response of bubbly viscoelastic media. The agreement with previous studies presents our new model as a valuable tool for the study of nonlinear ultrasound in bubbly soft tissues and materials.

## Highlights

• A nonlinear model coupling ultrasound with bubbles in viscoelastic media is derived.
• Nonlinear parameter β is computed to quantify acoustic nonlinear effects.
• The key role of elasticity on the nonlinear ultrasound propagation is emphasized.
• Shear elasticity strongly reduces harmonic generation more than viscous dissipation.
• Our model is useful to study nonlinear ultrasound in bubbly tissues.

## 1 Introduction

The propagation of acoustic waves in media containing microbubbles has attracted interest due to its relevant role in a variety of biomedical and industrial applications, such as ultrasound contrast imaging, targeted drug delivery [1], ultrasonic cleaning, and therapeutic ultrasound [2]. These applications exploit the complex, nonlinear dynamics of bubbles, which in turn introduce strong nonlinearities into the propagating pressure wave [3]. Such behavior not only generates higher harmonics and waveform distortion, but also contributes to energy dissipation and dispersion in the acoustic field, resulting in complex waveforms [4], [5].

Traditionally, Newtonian fluids, in which only viscosity is considered, have been the standard model in several studies of cavitation and wave dynamics [6], [7]. Extending this line of research, theoretical developments have led to the derivation of the Khokhlov–Zabolotskaya–Kuznetsov (KZK) equation for bubbly media, using volume-averaged formulations to account for the presence of multiple gas bubbles [8], [9], [10]. Within this context, significant contributions have been made by characterizing the acoustic field in bubbly liquids while incorporating essential physical effects such as nonlinearity, dispersion, and dissipation in one-, two-, and three-dimensional configurations [11], [12], [13].

In contrast, many biological tissues and synthetic materials of practical relevance exhibit viscoelastic behavior. In such media, the mechanical response involves both viscous damping and elastic stiffness, which strongly influence both wave propagation and bubble dynamics. Common rheological models used to describe viscoelastic materials include the Maxwell, Kelvin–Voigt, and Zener models [14]. Among these, the Kelvin–Voigt model [15] is often used as a first approximation, as it captures the elastic response of the medium while neglecting the effects of stress relaxation. Some soft tissues are frequently modeled as viscoelastic media that contains gas bubbles. Given that such tissues tend to return to their original configuration after deformation [16], Kelvin–Voigt-based models have gained increasing attention in recent years [15], [16], [17], [18], [19], [20], [21].

Generating a stable population of gas bubbles in a liquid medium is experimentally challenging, particularly under controlled acoustic excitation because of buoyancy and bubble agglomerations, and in the case of very large amplitude disturbances because of the breakup of the bubbles. This makes it difficult to obtain reproducible and stable experimental data in bubbly liquids. Some care had to be taken in the selection of a suitable liquid for experimental purposes. If the viscosity of the liquid is too small, the bubbles rise quickly through the liquid and only a mixture containing a very small volume of gas can be produced [22].

Given the challenges associated with experimental studies of cavitation, numerical modeling can provide insight into the physical effects produced on ultrasound and guide experiments. To account for the complex rheology of the aforementioned media, researchers have combined a number of non-Newtonian constitutive relationships, including viscoelastic, with Rayleigh–Plesset-like equations for bubble dynamics.

The dynamics of a single bubble that oscillates in such media have been extensively investigated [15], [17], [18], [23], [24], [25], [26], [27], [28], [29], [30]. These studies show that elasticity increases the pressure required to induce inertial oscillations, while the oscillations themselves become progressively damped as the elasticity is raised, indicating a strong stabilizing influence [30]. Experimental observations in hydrogels corroborate these findings, showing that increased shear modulus suppresses cavitation activity and limits bubble expansion [28]. Although tissue viscosity and elasticity do not significantly shift the onset of cavitation, they do affect the growth of bubbles [29]. Furthermore, greater elasticity and/or viscosity in the surrounding medium reduce the nonlinear dynamics of bubble oscillations [15], [19], [31].

Despite these advances, comprehensive models that describe nonlinear acoustic wave propagation in viscoelastic media containing multiple bubbles remain limited in scope. In particular, models that consistently couple bubble dynamics with the acoustic pressure field are still under development. Notably, Hasegawa et al. [32] incorporated shear elasticity into a weakly nonlinear one-dimensional wave equation, showing that elasticity reduces both acoustic nonlinearity and dissipation, while enhancing dispersion. Expanding on this framework, Kagami et al. [19] derived a KZK-type equation to describe the weakly nonlinear propagation of focused ultrasound in bubbly viscoelastic liquids. Their results demonstrate that shear elasticity suppresses the generation of higher harmonics and favors the persistence of the fundamental frequency, in contrast to the behavior observed in Newtonian liquids. These insights emphasize the importance of understanding how the constitutive properties of the medium affect nonlinear wave phenomena.

Acoustic waves of finite amplitude propagate nonlinearly in most fluids and solids [33]. Among these, bubbly liquids are especially notable because even a small volume fraction of gas bubbles can drastically modify the acoustic response of the medium. They exhibit significantly increased compressibility, enhanced dispersion, and a marked amplification of nonlinear effects [5], [34].

Understanding the propagation of nonlinear waves in these media requires careful quantification of the contributions of nonlinearity, dissipation, and dispersion [35]. A central quantity in this context is the nonlinear parameter β, which emerges from the Taylor expansion of the pressure–density relation under adiabatic conditions [36], [37], where A and B are the coefficients of the linear and quadratic terms, respectively:

(1) $$\beta =1+\frac{B}{2A}.$$

The ratio B/A (an alternative expression of the nonlinear contribution) accounts for the nonlinearity of the equation of state, while the additional term “1” reflects the convective contribution associated with particle velocity. The nonlinear parameter describes how a finite-amplitude acoustic wave progressively distorts as it propagates through the material. A higher value of β implies faster waveform distortion, making it a practical scalar for comparing the intrinsic nonlinearity of different media [5], [34]. In particular, bubbly liquids with elevated β values can exhibit pronounced nonlinear effects, such as harmonic generation and shock formation, even at relatively low acoustic intensities.

The nonlinear acoustic parameter β has been widely studied since the 1960s, when two main methods for its estimation were introduced: the thermodynamic and finite-amplitude approaches [36], [38], [39]. The thermodynamic method achieves high accuracy by measuring the sound speed under controlled pressure and temperature conditions, but it is less suitable for biological tissues, which may undergo structural changes upon heating [40]. The finite-amplitude method, in contrast, is experimentally simpler and generally preferred due to the possibility of simulating acoustic wave propagation [41].

In soft tissues, such as the liver, muscle, brain, and myocardium, reported β values typically range from 3.5 to 6.5, with higher values observed in fat-rich tissues, up to approximately 7.5 [37], [42]. These variations reflect differences in tissue composition and microstructure, supporting the use of β as a biomarker for tissue characterization and disease detection [43], [44], [45]. The structural dependencies of β have been investigated at tissue, cellular, and molecular scales, highlighting its sensitivity to pathological changes compared to other acoustic parameters [44], [46].

Despite its potential, clinical applications of this parameter remain limited. While studies in animals clearly differentiate between healthy and diseased tissues [44], investigations in human cancers have focused mainly on liver and breast [47]. Recent measurements in ex vivo kidney samples demonstrated that clear cell renal cell carcinoma exhibits significantly higher β and acoustic attenuation than healthy tissue. This increase is attributed to elevated lipid and glycogen content, consistent with previously reported high β values in fatty tissues. In biological solutions, β increases with protein concentration, as observed in hemoglobin and bovine serum albumin [48].

Tissue-mimicking phantoms composed of gelatin, agarose, or alginate show β values between 4.0 and 7.2 [49], comparable to those in biological soft tissues. Materials with well-characterized acoustic properties are crucial for fabricating tissue-mimicking phantoms to validate ultrasound imaging and for estimating thermal effects from nonlinear absorption in therapeutic ultrasound. In liquids, while β shows temperature dependence [50], [51], it is largely frequency independent within typical diagnostic ranges [52].

In bubbly media, gas bubbles drastically increase acoustic nonlinearity. At low bubble concentrations ($V_{vf}∼10^{-4}$), β can reach values up to $10^{4}$ [53], [54], far exceeding those of pure water (β≈5) or air (β≈0.4). Both theoretical and experimental works confirm that bubble dynamics, especially near resonance, dominates the nonlinear response in bubbly liquids [55]. For example, in a biphasic fluid of helium bubbles in Fluorinert FC-43, β was measured at 1380, compared to 7.6 in pure liquid [34]. Such elevated values highlight bubbly media as powerful nonlinear enhancers, with applications in parametric arrays, non-destructive testing, and contrast-enhanced imaging.

Nonetheless, there is a notable gap: no studies have reported β in viscoelastic media containing bubbles, such as tissue-like materials with embedded microbubbles. This combination is relevant for biomedical applications, including targeted drug delivery and ultrasound-mediated therapies, where viscoelasticity and nonlinear effects coexist. Quantifying β in these complex media would bridge the gap between tissue characterization and nonlinear acoustics. Comparison of the nonlinearity of different materials can thus provide valuable insight in selecting optimal media with appropriate rheological properties for such studies.

In this work, we derive a model that governs the propagation of nonlinear pressure waves in viscoelastic media that explicitly accounts for the elasticity of the medium. To our knowledge, no differential system combining the Rayleigh–Plesset equation written in terms of bubble volume variation with the wave equation has yet been proposed to describe nonlinear ultrasound in viscoelastic media containing bubbles. This study aims to investigate how fluid rheology influences the ultrasonic behavior in such media.

Section 2 presents the mathematical formulation that couples the propagation of ultrasonic waves with bubble dynamics through the use of the variation of bubble volume as a dependent variable of bubble dynamics. This framework allows simultaneous tracking of changes in the acoustic field and bubble volume over time, as demonstrated in Section 3.1, where the influence of the pressure source amplitude on harmonic generation and the effect of shear elasticity are also examined. A law is proposed for the description of the second and third harmonic amplitudes as functions of the acoustic pressure amplitude at the source by varying the elasticity of the medium. In Section 3.2, the nonlinear propagation of finite-amplitude ultrasound is analyzed, and the nonlinear acoustic parameter is evaluated for a set of representative soft media. The effects of elasticity and viscosity are also studied. Finally, conclusions are provided in Section 4.

## 2 Material and methods

This section presents the physical problem we consider here, related to the nonlinear interaction of ultrasound and gas bubbles in a viscoelastic medium, the mathematical model derived and used to couple the acoustic pressure field with the volume oscillations of bubbles in a viscoelastic medium, and the numerical model developed to solve it. Building on the framework in [3], we introduce a modified Rayleigh–Plesset equation expressed in terms of bubble volume variation, incorporating the Kelvin–Voigt model to capture the linear viscoelastic response of the surrounding medium [30]. This formulation enables the study of nonlinear ultrasonic wave propagation in bubbly viscoelastic media through numerical simulations obtained from the development of an algorithm based on the finite-volume and finite-difference methods, in space and time, respectively.

### 2.1 Physical problem and mathematical model

We consider a resonator of length L filled with a bubbly viscoelastic fluid. We assume a homogeneous monodisperse distribution of spherical gas bubbles in an isotropic viscoelastic medium. The nonlinear interaction between the acoustic waves and the bubble oscillations is described by a differential system formed by the wave equation, Eq. (2), and a Rayleigh–Plesset equation combined with the linear viscoelastic Kelvin–Voigt model, Eq. (3), to account for the viscous and elastic response of the surrounding medium. For detailed derivations of Eq. (3), we refer the reader to [30]. The system couples the acoustic pressure p(x,t) and the bubble volume variation $v\left(x,t\right)=V\left(x,t\right)-V_{0}$, where V(x,t) is the current bubble volume, $V_{0}=\frac{4}{3}\pi R_{0}^{3}$ is the initial bubble volume, $R_{0}$ is the initial bubble radius, x is the one-dimensional space coordinate, and t denotes the time [5], [56],

(2) $$\frac{\partial^{2}p}{\partial x^{2}}-\frac{1}{c_{0}^{2}}\frac{\partial^{2}p}{\partial t^{2}}=-\rho N_{g}\frac{\partial^{2}v}{\partial t^{2}},\;0<x<L,\;0<t<T,$$

(3) $$\frac{\partial^{2}v}{\partial t^{2}}+\delta \omega_{b}\frac{\partial v}{\partial t}+\left(\omega_{b}^{2}+\bar{G}\right)v+\eta p=av^{2}+b\left(2v\frac{\partial^{2}v}{\partial t^{2}}+{\left(\frac{\partial v}{\partial t}\right)}^{2}\right),$$

0≤x≤L,0<t<T.

In Eq. (2), $c_{0}$ is the sound speed and ρ is the density at the equilibrium state, and $N_{g}$ is the bubble density in the medium. In Eq. (3), $\delta =4\mu /\rho \omega_{b}R_{0}^{2}$ is the viscous damping coefficient of the bubbly medium [57] with μ the dynamic viscosity of the medium, $\bar{G}=4G/\left(\rho R_{0}^{2}\right)$ is the elastic coefficient of the medium [30], with G the shear modulus (or rigidity) of the viscoelastic medium, $\omega_{b}^{2}=3\gamma P_{b0}/\left(\rho R_{0}^{2}\right)$ is the bubble resonance frequency in a Newtonian fluid [14], where γ is the ratio of the specific heat capacities of the gas, $P_{b0}=\rho_{b}c_{b}^{2}/\gamma$ is the atmospheric pressure of the gas, and $\rho_{b}$ and $c_{b}$ are the density and speed of sound in the equilibrium state of the gas. Note that the elastic contribution vanishes as G→0 and only the viscous stress remains, thereby recovering the classical Rayleigh–Plesset equation for a Newtonian liquid. The parameter $\eta =4\pi R_{0}/\rho$ is a constant. The nonlinear coefficients related to the adiabatic gas law and accounting for the dynamic response of bubbles are given by $a=\omega_{b}^{2}\left(\gamma +1\right)/\left(2V_{0}\right)$ and $b=1/\left(6V_{0}\right)$, respectively.

In this work, we adopt the Kelvin–Voigt model as a first approximation, owing to its simplicity and its ability to capture the elastic recovery behavior typically observed in soft tissues. Recent studies have also demonstrated its effectiveness in qualitatively reproducing bubble dynamics under similar conditions. Several other linear viscoelastic formulations, such as the Maxwell and Zener models, are commonly employed to simulate bubble oscillations in soft media. Future work will explore the Zener model, which introduces both elasticity and a relaxation time, providing a more complete representation of memory effects and delayed recovery in viscoelastic media.

The model assumes that dispersion and nonlinear phenomena are exclusively due to the presence of bubbles. It considers the following physical restrictions: bubbles oscillate at their first radial mode, surface tension is neglected, they pulsate at finite but moderate amplitude, thus collapse is not modeled; they do not radiate sound themselves, the adiabatic gas law is used, and their thermal damping is considered negligible. In addition, void fraction in the medium must be much lower than one, and bubble-bubble interactions, buoyancy, Bjerknes forces, and viscous drag are not included.

We impose the following initial conditions, assuming that both p and v are at rest at the outset:

(4) p(x≠0,0)=0,v(x,0)=0,

$$\frac{\partial p}{\partial t}\left(x\neq 0,0\right)=0,\;\frac{\partial v}{\partial t}\left(x,0\right)=0,\;0\leq x\leq L.$$

To excite the system, a time-dependent pressure source of frequency f and amplitude $p_{0}$ is applied at x=0, leading to the boundary condition:

(5) $$p\left(0,t\right)=p_{0}\sin\left(2\pi ft\right),\;0\leq t\leq T.$$

Two types of resonators are considered, each with a specific boundary condition at x=L: the free-walled cavity,

(6) p(L,t)=0,0≤t≤T,

and the open-field cavity,

(7) $$\frac{\partial p}{\partial x}\left(L,t\right)=-\frac{1}{c_{0}}\frac{\partial p}{\partial t},\;0\leq t\leq T.$$

The validity of the model is limited to cases where the bubble volume variation ($v\ll V_{0}$), meaning that bubble oscillations remain of moderate amplitude. If the acoustic pressure $p_{0}$ exceeds a certain threshold, this assumption is no longer valid, and the model may not accurately capture the bubble dynamics. In all simulations reported here, this condition is satisfied.

### 2.2 Numerical model

Both differential systems, Eqs. (2)–(6) for the free-walled cavity, on the one hand, Eqs. (2)–(5), (7) for the open-field cavity, on the other hand, are solved using the numerical model based on the finite-difference method in the time domain and the finite-volume method in the space dimension, developed here.

#### 2.2.1 Approximation in the time domain (finite-difference method)

**Fig. 1:**
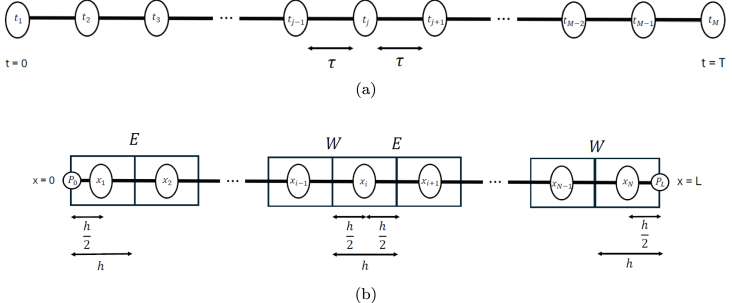
(a) Finite-difference discretization of the time domain (subscript j). (b) Finite-volume discretization of the space domain (subscript i, control volume of center $x_{i}$).

The time domain T is divided into M−1 constant intervals of duration τ. Each discretization point in time is denoted by $t_{j}$ (j=2,…,M−1). The following equations are used to approximate the time derivatives that appear in Eqs. (2)–(4) (Fig. 1(a)):

(8) $$\frac{\partial^{2}p_{j}}{\partial t^{2}}\approx \frac{p_{j+1}-2p_{j}+p_{j-1}}{\tau^{2}},\;\frac{\partial^{2}v_{j}}{\partial t^{2}}\approx \frac{v_{j+1}-2v_{j}+v_{j-1}}{\tau^{2}},$$

(9) $$\frac{\partial p_{j}}{\partial t}\approx \frac{p_{j}-p_{j-1}}{\tau },\;\frac{\partial v_{j}}{\partial t}\approx \frac{v_{j}-v_{j-1}}{\tau }.$$

The error in these approximations is $O\left(\tau^{2}\right)$ for the second-order derivatives and O(τ) for the first-order derivatives [58], [59].

#### 2.2.2 Approximation in the space dimension (finite-volume method)

The length of the resonator L is divided into N control volumes $V_{C}$ of length h. The central point of each $V_{C}$ is denoted by $x_{i}$ (i=1,…,N). The differential equations are integrated in each $V_{C}$. The following equations are used to approximate the integrals that come from Eq. (2), where E and W indicate the east and west sides of $V_{C}$, respectively, and K is a generic constant (Fig. 1(b)):

(10) $$\int_{V_{C}}\frac{\partial^{2}p_{i}}{\partial x^{2}}\;dv=\int_{W}^{E}\frac{\partial^{2}p_{i}}{\partial x^{2}}\;dx={\left(\frac{\partial p}{\partial x}\right|}_{E}-{\left(\frac{\partial p}{\partial x}\right|}_{W}\approx \frac{p_{i+1}-p_{i}}{h}-\frac{p_{i}-p_{i-1}}{h}=\frac{p_{i+1}-2p_{i}+p_{i-1}}{h},$$

(11) $$\int_{V_{C}}K\;dv=\int_{W}^{E}K\;dx=Kh.$$

The error in the approximation used in Eq. (10) is O(h) [58], [60].

The application of Eqs. (8)–(10) to Eqs. (2), (3) is described below. The first and last control volumes must consider specific applications of Eq. (10) to Eq. (2).

#### 2.2.3 Generic volume

For any $t_{j}$ (j=2,…,M−1) and any control volume $V_{C}$ (i=2,…,N−1), Eq. (1) yields, after multiplying by $h\tau^{2}$:

(12) $$\frac{h^{2}}{c_{0}^{2}}p_{i,j+1}-\rho N_{g}h^{2}v_{i,j+1}=\tau^{2}p_{i+1,j}+\tau^{2}p_{i-1,j}+2\left(\frac{h^{2}}{c_{0}^{2}}-\tau^{2}\right)p_{i,j}-\frac{h^{2}}{c_{0}^{2}}p_{i,j-1}\;-2\rho N_{g}h^{2}v_{i,j}+\rho N_{g}h^{2}v_{i,j-1},\;j=2,\ldots ,M-1;\;i=2,\ldots ,N-1.$$

After multiplying by $\tau^{2}$, Eq. (3) yields:

(13) $$\left(2bv_{i,j}-1\right)v_{i,j+1}=\left(1-\delta \omega_{b}\tau -bv_{i,j-1}\right)v_{i,j-1}+\left(-2+\delta \omega_{b}\tau +\tau^{2}\left(\omega_{b}^{2}+\bar{G}\right)\right)v_{i,j}\;+\eta \tau^{2}p_{i,j}+\left(-a\tau^{2}+3b\right)v_{i,j}^{2},\;j=2,\ldots ,M-1;\;i=2,\ldots ,N-1.$$

#### 2.2.4 First volume

The approximation for i=1 is (Fig. 1(b)):

(14) $$\int_{V_{C}}\frac{\partial^{2}p_{1}}{\partial x^{2}}\;dv=\int_{W}^{E}\frac{\partial^{2}p_{1}}{\partial x^{2}}\;dx={\left(\frac{\partial p}{\partial x}\right|}_{E}-{\left(\frac{\partial p}{\partial x}\right|}_{W}\approx \frac{p_{2}-p_{1}}{h}-\frac{p_{1}-P_{0}}{h/2}=\frac{p_{2}-3p_{1}+2P_{0}}{h},$$

where $P_{0}$ is the pressure at x=0 (see Eq. (5)). Thus, Eq. (2) for the first volume yields, after multiplying by $h\tau^{2}$:

(15) $$\frac{h^{2}}{c_{0}^{2}}p_{1,j+1}-\rho N_{g}h^{2}v_{1,j+1}=\tau^{2}p_{2,j}+2\tau^{2}P_{0}+\left(2\frac{h^{2}}{c_{0}^{2}}-3\tau^{2}\right)p_{1,j}\;-\frac{h^{2}}{c_{0}^{2}}p_{1,j-1}-2\rho N_{g}h^{2}v_{1,j}+\rho N_{g}h^{2}v_{1,j-1},\;i=1;\;j=2,\ldots ,M-1.$$

#### 2.2.5 Last volume

Since two different boundary conditions are considered at x=L, we derive two distinct approximations for the last control volume i=N (Fig. 1(b)).

In the case of a free-walled cavity, defined by Eq. (6), the spatial second derivative of pressure over the control volume yields:

(16) $$\int_{V_{C}}\frac{\partial^{2}p_{N}}{\partial x^{2}}\;dv=\int_{W}^{E}\frac{\partial^{2}p_{N}}{\partial x^{2}}\;dx={\left(\frac{\partial p}{\partial x}\right|}_{E}-{\left(\frac{\partial p}{\partial x}\right|}_{W}\approx \frac{P_{L}-p_{N}}{h/2}-\frac{p_{N}-p_{N-1}}{h}=\frac{2P_{L}-3p_{N}+p_{N-1}}{h},$$

where $P_{L}$ denotes the pressure at x=L (see Eq. (6)). Substituting this approximation into Eq. (2) and multiplying through by $h\tau^{2}$, the discrete equation for the last volume becomes:

(17) $$\frac{h^{2}}{c_{0}^{2}}p_{N,j+1}-\rho N_{g}h^{2}v_{N,j+1}=2\tau^{2}P_{L}+\tau^{2}p_{N-1,j}+\left(2\frac{h^{2}}{c_{0}^{2}}-3\tau^{2}\right)p_{N,j}\;-\frac{h^{2}}{c_{0}^{2}}p_{N,j-1}-2\rho N_{g}h^{2}v_{N,j}+\rho N_{g}h^{2}v_{N,j-1},\;i=N;\;j=2,\ldots ,M-1.$$

On the other hand, for the open-field cavity, defined by Eq. (7), the boundary condition at x=L leads to the following approximation:

(18) $$\int_{V_{C}}\frac{\partial^{2}p_{N}}{\partial x^{2}}\;dv=\int_{W}^{E}\frac{\partial^{2}p_{N}}{\partial x^{2}}\;dx={\left(\frac{\partial p}{\partial x}\right|}_{E}-{\left(\frac{\partial p}{\partial x}\right|}_{W}\approx -\frac{1}{c_{0}}\frac{\partial p}{\partial t}-\frac{p_{N}-p_{N-1}}{h}\approx -\frac{1}{c_{0}}\frac{p_{N,j}-p_{N,j-1}}{\tau }-\frac{p_{N}-p_{N-1}}{h}.$$

Inserting this into Eq. (2) and multiplying by $h\tau^{2}$, we obtain the discrete expression for the last volume in the open-field configuration:

(19) $$\frac{h^{2}}{c_{0}^{2}}p_{N,j+1}-\rho N_{g}h^{2}v_{N,j+1}=\tau^{2}p_{N-1,j}+\left(\frac{-\tau h}{c_{0}}-\tau^{2}+2\frac{h^{2}}{c_{0}^{2}}\right)p_{N,j}+\;\left(\frac{\tau h}{c_{0}}-\frac{h^{2}}{c_{0}^{2}}\right)p_{N,j-1}-2\rho N_{g}h^{2}v_{N,j}+\rho N_{g}h^{2}v_{N,j-1},i=N;\;j=2,\ldots ,M-1.$$

Therefore, Eqs. (12), (13), (15), and (17) define a complete system for the free-walled cavity, while Eqs. (12), (13), (15), and (19) correspond to the open-field cavity case. These equations are solved in the entire space domain at each time step. In the end, we obtain the values of the acoustic pressure and bubble volume variation in the entire resonator at any time. In particular, the open-field configuration is employed for the evaluation of the nonlinear parameter β in Section 3.2.

## 3 Results

This section presents numerical simulations carried out by using the model introduced in Section 2, focusing on the propagation of nonlinear ultrasound in bubbly viscoelastic media. In Section 3.1, we analyze the influence of bubble oscillations and medium elasticity on the acoustic field in a cavity under free-walled boundary conditions. A comparison between linear and nonlinear regimes is provided in Section 3.1.1 for a set of representative soft media, while the spatial distribution of harmonic components is examined in Section 3.1.2. Additionally, a predictive law is proposed for the second- and third-harmonic amplitudes as functions of the source amplitude for a given shear modulus. Section 3.2 investigates the acoustic nonlinearity parameter β in representative soft media using a finite-amplitude method based on harmonic generation in a cavity in an open-field configuration. Finally, the influence of viscoelasticity on β is assessed. Simulations assume stable gas bubbles with radius $R_{0}=4.5\;\mu \text{m}$, and gas properties $c_{b}=340\;\mathrm{m/s}$ and $\rho_{b}=1.29\;{\mathrm{kg/m}}^{3}$. In all cases, a steady state is reached within the resonator. Note that the situation assumed in the numerical simulations corresponds to the presence of stable oscillating bubbles that could be experimentally produced by a microbubble generator introducing gas into the fluid through a porous material, by using ultrasound contrast agent suspensions, or through controlled techniques such as microinjection, laser excitation, stable acoustic cavitation, or hydrodynamic cavitation.

### 3.1 Wave propagation in bubbly viscoelastic materials

In this section, we present the temporal evolution of acoustic pressure waves in different media by comparing waveforms in water [3], a 6 wt% gelatin gel [20], and liver tissue [19]. The rheological properties of these media (density ρ, viscosity μ, and shear modulus G) are listed in Table 1.

The bubble density is $N_{g}=2\times 10^{11}\;m^{-3}$, corresponding to a volumetric void fraction $V_{vf}=0.0076\text{\%}$, and the driving frequency is set at f=200kHz. The sound speed in the bubbly media, $c_{bf}$, is determined from the properties $N_{g}$, $R_{0}$ and the driving frequency f. For bubbly viscoelastic media, it is computed from simulations under open-field conditions, while for the liquid with bubbles it follows the procedure of Hamilton and Blackstock [5]. Table 2 reports the sound speeds in both the homogeneous and bubbly media.

**Table 1:** Rheological properties of considered viscoelastic media. Water is included for comparison.

Medium	Density ρ (kg/m${}^{3}$)	Viscosity μ (mPa s)	Shear modulus G (kPa)
Water	1000	1	0

6 wt% gelatin gel	1020	18.3	4

Liver	1100	9	40

**Table 2:** Physical properties of different media.

Medium	$c_{0}$ (m/s)	$c_{bf}$ (m/s)
Water	1500	1002.2
Gelatin 6% wt	1567	1037
Liver	1549	1080

We use our model with the parameter values employed in [3]. We consider a total simulation time of T=50P, where P=1/f is the acoustic period. In this section, we use the time step $\tau =1.25\times 10^{-8}\;s$, which corresponds to 400 points per period, and the spatial length of the control volumes $h=1.96\times 10^{-5}\;m$, which corresponds to 256 control volumes per wavelength. These values ensure the convergence of the numerical solution. The length of the resonant cavity is set at L=λ/4, where $\lambda =c_{bf}/f$ is the wavelength at frequency f in the bubbly viscoelastic medium.

Simulations are performed using the configuration given by Eqs. (12), (13), (15), and (17) for the free-walled case.

#### 3.1.1 Comparison between linear and nonlinear regimes

We compare the acoustic responses of the system under two excitation amplitudes: $p_{0}=1\;\text{mPa}$ and $p_{0}=28\;\text{kPa}$, corresponding, respectively, to the linear and nonlinear regimes.

**Fig. 2:**
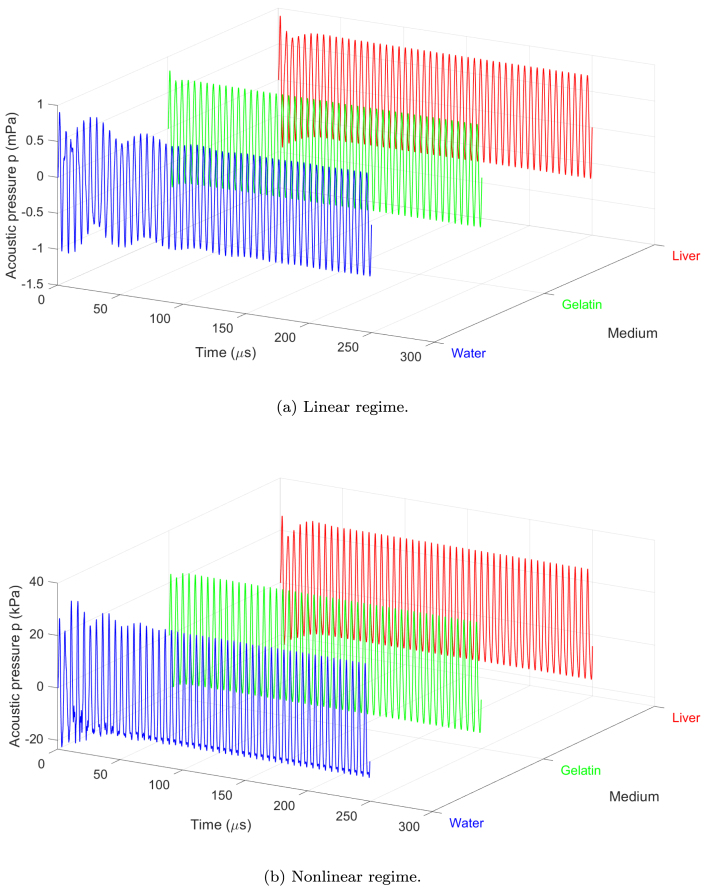
Waveform at L/2 during 0≤t≤T in the bubbly media. (a) Infinitesimal amplitude $p_{0}=1\;\text{mPa}$ (linear regime), (b) finite amplitude $p_{0}=28\;\text{kPa}$ (nonlinear regime). $R_{0}=4.5\;\mu \text{m}$, $N_{g}=2\times 10^{11}\;m^{-3}$, f=200kHz, L=λ/4.

**Fig. 3:**
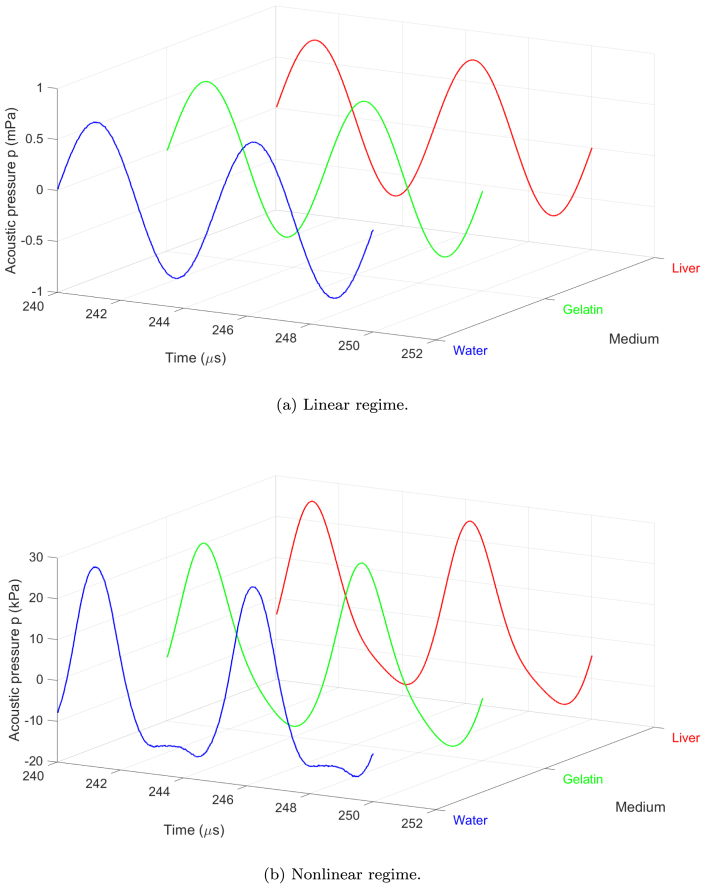
Waveform at L/2 during two periods in the bubbly media. (a) Infinitesimal amplitude $p_{0}=1\;\text{mPa}$ (linear regime), (b) finite amplitude $p_{0}=28\;\text{kPa}$ (nonlinear regime). $R_{0}=4.5\;\mu \text{m}$, $N_{g}=2\times 10^{11}\;m^{-3}$, f=200kHz, L=λ/4.

Fig. 2 shows the temporal evolution of the acoustic pressure at the midpoint of the resonator x=L/2 over the full simulation time for both amplitudes. To better visualize the details of the waveform, Fig. 3 displays a zoom over two representative periods in the steady state.

The results reveal a clear transition from sinusoidal waveforms in the linear regime to asymmetric and steepened waveforms in the nonlinear regime, characteristic of nonlinear acoustic propagation. In the linear case, the pressure response remains proportional to the input amplitude and no significant differences are observed in the maximum pressure amplitude across the three media studied.

In the nonlinear regime, waveform distortion becomes pronounced due to nonlinear bubble oscillations. The bubbly liquid exhibits the earliest and most intense nonlinear distortion, characterized by steepening of the waveform. This is due to the lower mechanical resistance of the medium, allowing more energy to be transferred to nonlinear components. However, gelatin and liver media, which have higher shear modulus and viscosity, attenuate nonlinear effects by damping nonlinear bubble oscillations and absorbing acoustic energy. This leads to a reduction in the amplitude of positive pressure peaks and a loss of waveform symmetry, delaying and weakening nonlinear distortion. These findings emphasize the role of medium viscoelastic properties in modulating nonlinear acoustic phenomena and suggest implications for the design and optimization of ultrasonic applications in soft tissues.

#### 3.1.2 Influence of source amplitude and elasticity on harmonic generation

The generation of the second and third harmonics of acoustic pressure is analyzed as a function of the variation of the source amplitude $p_{0}$ and the shear modulus G to determine the conditions that influence its harmonic content.

**Fig. 4:**
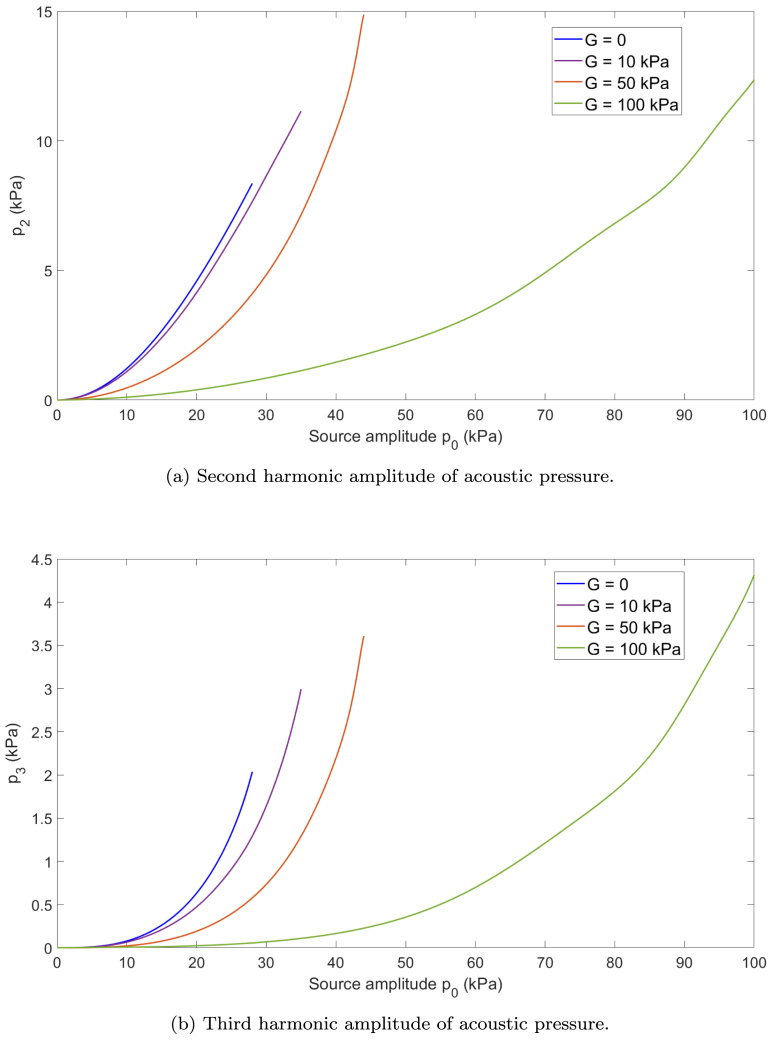
(a) Second and (b) third harmonic amplitudes of acoustic pressure vs. source amplitude $p_{0}$ in the bubbly media. $R_{0}=4.5\;\mu \text{m}$, $N_{g}=2\times 10^{11}\;m^{-3}$, f=200kHz, L=λ/4.

To visualize how shear elasticity affects harmonic generation, the amplitudes of the second and third harmonics are calculated for different excitation amplitudes at the source, as shown in Fig. 4. The shear modulus is varied in the following range of values, G=0,10,50,100kPa, representative of soft biological tissues [29]. For all cases, viscosity and density are fixed to those of water, μ=0.0014Pas and $\rho =1000\;{\text{kg/m}}^{3}$, to isolate the effect of elasticity.

The numerical model developed in this study enables the use of higher pressure amplitudes at the source when simulating media with increased stiffness. Specifically, maximum source amplitudes of $p_{0}=28,35,44$, and 100kPa are used for each viscoelastic configuration, respectively.

Fig. 4 shows the maximum harmonic amplitudes obtained after applying a Fast Fourier Transform (FFT) to the pressure signals. As the excitation amplitude increases, the amplitudes of all harmonic components also increase, as previously observed in [3].

However, in viscoelastic media, increasing the stiffness leads to a noticeable attenuation of the second and third harmonics. This effect is evidenced by the smoother growth in the harmonic amplitude curves as the shear modulus increases. Harmonic generation occurs only when the input pressure amplitude is sufficiently high to trigger nonlinear behavior in the bubbly medium. Significant amplitude values of pressure harmonic can only be observed once a threshold is reached. As the shear modulus increases, the medium becomes stiffer, and consequently higher source amplitudes are required to induce significant nonlinear effects. This trend is reflected in the shift of the curves and the thresholds to the right as the shear modulus increases. Specifically, for lower values of G, such as 0 or 20 kPa, the harmonic amplitudes grow rapidly with increasing $p_{0}$. In contrast, for stiffer media with G=50 or 100kPa, comparable harmonic amplitudes are only reached at substantially higher excitation levels. This behavior demonstrates how increasing stiffness suppresses harmonic generation, as reported in [19], unless compensated by a stronger acoustic excitation. Moreover, the decrease in nonlinearity due to shear elasticity is qualitatively consistent with the previous result [30] that focuses on the behavior of a single bubble.

Table 3, Table 4 present the polynomial fitting expressions of the amplitude of the second and third harmonics of acoustic pressure, respectively, as functions of the input pressure amplitude $p_{0}$ (in kPa). These expressions provide a convenient way to characterize the nonlinear response of the system under varying excitation levels and different viscoelastic properties of the surrounding medium.

**Table 3:** Polynomial fitting of the second harmonic amplitude of acoustic pressure as a function of source pressure $p_{0}$.

Shear modulus G (kPa)	Fitting expression $p_{2}\;\left(p_{0}\right)$
G=0	$p_{2}=-0.0001118\;p_{0}^{3}+0.0141\;p_{0}^{2}$ $-0.008981\;p_{0}+0.01141$

G=10	$p_{2}=-0.00010233\;p_{0}^{3}+0.013117\;p_{0}^{2}$ $-0.014999\;p_{0}+0.029049$

G=50	$p_{2}=0.00020291\;p_{0}^{3}-0.0039173\;p_{0}^{2}$ $+0.10348\;p_{0}-0.26065$

G=100	$p_{2}=1.0838\times 10^{-5}\;p_{0}^{3}-0.00010673\;p_{0}^{2}$ $-0.025066\;p_{0}-0.11022$

**Table 4:** Polynomial fitting of the third harmonic amplitude of acoustic pressure as a function of source pressure $p_{0}$.

Shear modulus G (kPa)	Fitting expression $p_{3}\;\left(p_{0}\right)$
G=0	$p_{3}=0.00014198\;p_{0}^{3}-0.0020266\;p_{0}^{2}$ $+0.016683\;p_{0}-0.027536$

G=10	$p_{3}=0.00011297\;p_{0}^{3}-0.0023162\;p_{0}^{2}$ $+0.025944\;p_{0}-0.055922$

G=50	$p_{3}=8.9336\times 10^{-5}\;p_{0}^{3}-0.0030286\;p_{0}^{2}$ $+0.037514\;p_{0}-0.096382$

G=100	$p_{3}=7.2375\times 10^{-6}\;p_{0}^{3}-0.00040157\;p_{0}^{2}$ $-0.0099394\;p_{0}-0.051946$

**Fig. 5:**
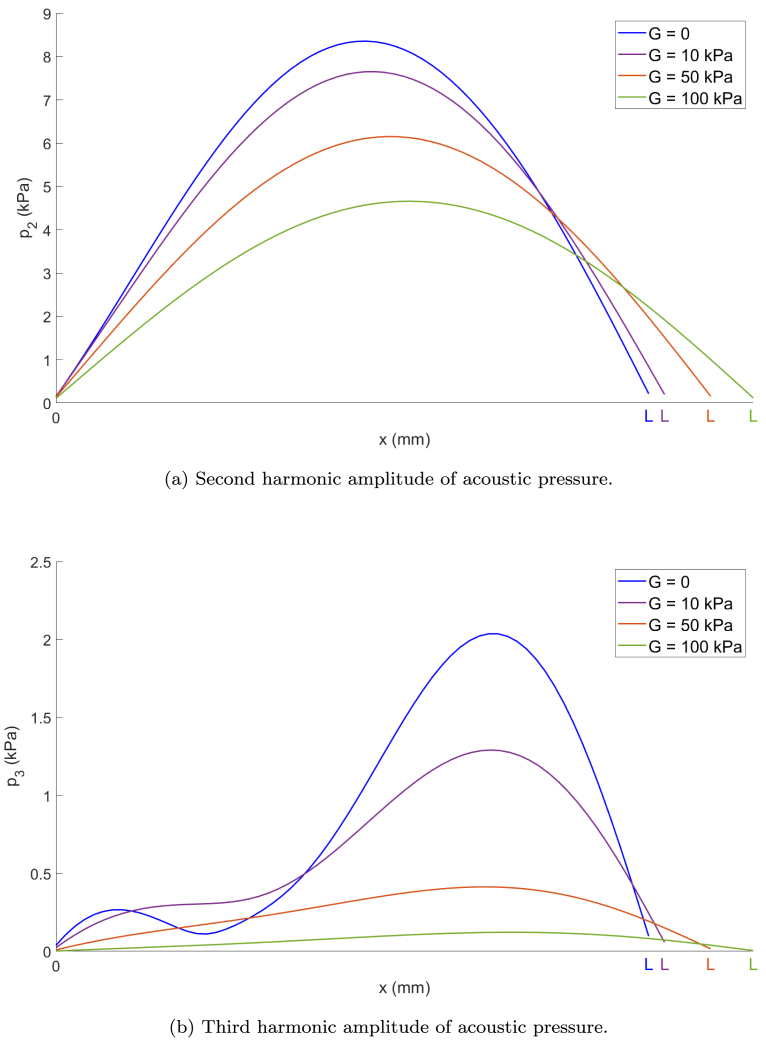
Distribution of (a) second and (b) third harmonic amplitude of acoustic pressure in the bubbly media. $R_{0}=4.5\;\mu \text{m}$, $N_{g}=2\times 10^{11}\;m^{-3}$, $p_{0}=28\;\text{kPa}$, f=200kHz, L=λ/4.

Lastly, keeping the source amplitude $p_{0}$ fixed, Fig. 5 shows the spatial distribution of the second harmonic component of the acoustic pressure (Fig. 5(a)) and the third harmonic component of the acoustic pressure (Fig. 5(b)) throughout the entire resonator for the highest common amplitude, $p_{0}=28\;\text{kPa}$, obtained by via FFT from x=0 to x=L.

Although the excitation is identical, the harmonic responses vary significantly depending on the medium. For both harmonics, the maximum amplitudes are consistently observed within the bubbly liquid region. This is attributed to the absence of elasticity in the bubbly medium, which allows more efficient energy transfer to the harmonic components. Consequently, nonlinear propagation and harmonic distortion arise even at lower source intensities in the bubbly liquid. In contrast, elastic media dampen these nonlinear effects, reducing harmonic amplitudes due to the restorative forces present, as noted before.

It is also worth noting that the spatial profiles of the second and third harmonics do not precisely correspond to half- and three-quarter wavelengths, respectively, due to the dispersive nature of sound speed in bubbly media.

The analysis of harmonic content highlights the crucial role of medium elasticity in the development and attenuation of nonlinear components, particularly in bubbly liquids where elasticity is absent. This motivates a more quantitative characterization of the nonlinear behavior of bubbly viscoelastic media.

### 3.2 Nonlinear parameter in bubbly viscoelastic media

The acoustic nonlinearity parameter β, which quantifies the nonlinear response of a material to acoustic excitation, is numerically evaluated for bubbly viscoelastic media using a variation of the finite-amplitude method, as described in [61]. The finite-amplitude method has been successfully applied to estimate β in homogeneous liquids, viscoelastic materials, and bubbly liquids. In the present study, we extend this methodology to the case of bubbly viscoelastic media, where both viscoelasticity and bubble dynamics contribute to the nonlinear acoustic behavior.

This approach relates β to the ratio of the second harmonic amplitude of the acoustic pressure, $p_{2}\left(x\right)$, to the square of the amplitude of the fundamental frequency component of the acoustic pressure, $p_{1}\left(x\right)$ at the driving frequency f. The growth of $p_{2}\left(x\right)$ relative to $p_{1}\left(x\right)$ quantifies the progressive distortion of the wave as it travels through the bubbly medium. Both quantities are obtained by applying a FFT to the simulated time-domain pressure signal and extracting the spectral amplitude at the corresponding frequency. These values of $p_{1}\left(x\right)$ and $p_{2}\left(x\right)$ are evaluated at various distances x from the acoustic source using the numerical model introduced in Section 2.2 under the open-field configuration given by Eqs. (12), (13), (15), and (19).

To ensure the validity of the method, excitation pressures are carefully selected to minimize the generation of higher-order harmonics (third and above), which could compromise the applicability of the finite-amplitude method employed here. The source amplitudes are chosen so that the second harmonic is clearly observable while the contribution of the third harmonic remains negligible.

Following the approach of [61], the nonlinear parameter β can be estimated from the linear growth of the ratio $p_{2}\left(x\right)/p_{1}^{2}\left(x\right)$ with distance. This relationship is expressed as:

(20) $$\text{slope}{\left(\frac{p_{2}}{p_{1}^{2}}\right)|}_{x}=\frac{\beta \pi f}{\rho_{bf}c_{bf}^{3}}x,$$

where $\rho_{bf}$ and $c_{bf}$ are the density and sound speed of the bubbly medium at frequency f. Here, the slope S of the linear fit is directly proportional to the acoustic nonlinearity parameter β, which is given by:

(21) $$\beta =\frac{S\rho_{bf}c_{bf}^{3}}{\pi f}.$$

Simulations are performed in an open-field configuration, as described in Section 2.2, with bubbles uniformly distributed at a density of $N_{g}=1\times 10^{11}\;{\text{bubbles/m}}^{3}$, corresponding to a volumetric void fraction $V_{vf}=0.0038\text{\%}$. The system is excited at a frequency of f=20kHz. As in Section 3.1, the sound speed in the bubbly media, $c_{bf}$, is computed from simulations for viscoelastic media under open-field conditions, and for liquid with bubbles following [5].

First, we compute the nonlinear parameter β for the viscoelastic media listed in Table 1. We next investigate the effects of medium elasticity and viscosity on the nonlinear behavior of the bubbly fluid.

#### 3.2.1 Determination of the nonlinear parameter in bubbly representative soft viscoelastic media

The numerical results presented in Section 3.1 reveal the nonlinear behavior of ultrasound at relatively high amplitudes in the media listed in Table 1. In this section, we evaluate the nonlinear parameter β of these bubbly soft viscoelastic media applying the approach described above.

The amplitude values of the fundamental and second harmonic of acoustic pressure are first calculated. As mentioned before, source amplitudes are chosen to minimize higher-order harmonic generation. These values, together with the corresponding sound speeds in the bubbly media, are reported in Table 5.

**Table 5:** Sound speed in the bubbly medium, $c_{bf}$, at frequency f=20 kHz for bubbles with radius $R_{0}=4.5\;\mu \text{m}$ and density $N_{g}=1\times 10^{11}\;m^{-3}$, and source amplitude $p_{0}$ suitable for the calculation of the nonlinear parameter β for the media listed in Table 1.

Medium	Sound speed $c_{bf}$ (m/s)	Source amplitude $p_{0}$ (Pa)
Water	1194,8	2300
6 wt% gelatin gel	1232,1	3400
Liver	1269,3	5100

**Fig. 6:**
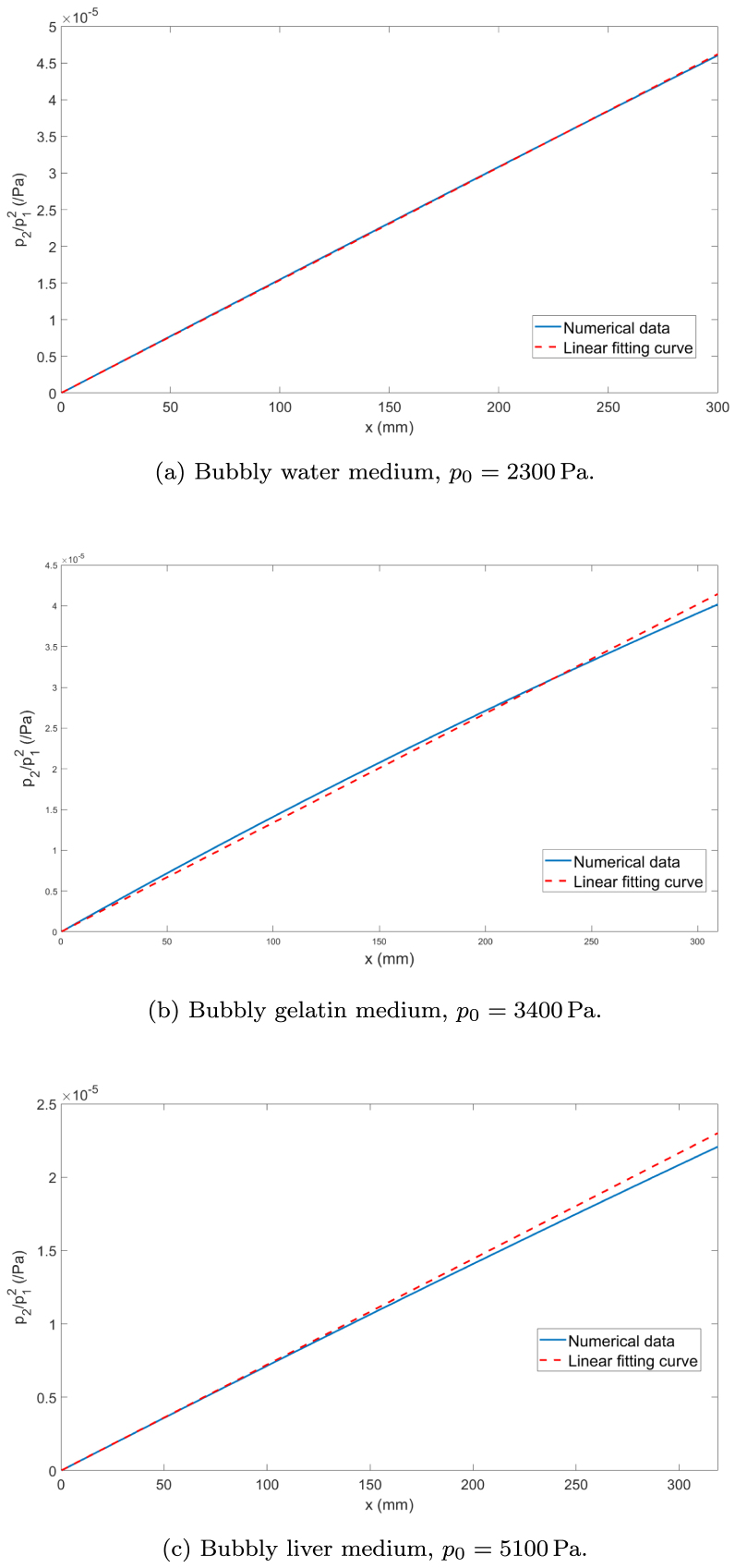
Ratio of the second harmonic amplitude of the acoustic pressure, $p_{2}\left(x\right)$, to the square of the amplitude of the fundamental frequency component of the acoustic pressure versus distance from the source. $R_{0}=4.5\;\mu \text{m}$, $N_{g}=1\times 10^{11}\;m^{-3}$, f=20kHz. (a) Bubbly water medium. (b) Bubbly gelatin medium. (c) Bubbly liver medium.

Figs. 6(a), 6(b), and 6(c) present the second harmonic amplitude, $p_{2}$, as a function of the square of the fundamental harmonic, $p_{1}^{2}$, measured up to a distance of x=300mm from the source. The figures also include linear fits corresponding to the water, gelatin, and liver media, respectively.

For water, the slope of the linear fit is S=0.00015396, corresponding to a nonlinear parameter β=4179.2. In the case of gelatin, the slope decreases slightly to S=0.00013393, with a corresponding β value of 4062.6. For the liver, the slope is further reduced to $S=7.2152\times 10^{-5}$, yielding a nonlinear parameter β=2580.8.

These values of nonlinear parameter are several orders of magnitude higher than the β values typically measured in homogeneous media, which range from 5 to 12 [42], [49]. In particular, the medium exhibiting the lowest β value is also the one with the highest shear modulus, which motivates an investigation of the influence of elasticity in the next section.

#### 3.2.2 Effects of medium elasticity on the nonlinear parameter

To further investigate the influence of elasticity on acoustic nonlinearity, we now extend this analysis to four biological soft tissues listed in Table 6, by using their characteristic shear modulus values as reported in [32], while maintaining the same reference values for density $\rho =1000\;{\text{kg/m}}^{3}$, sound speed $c_{0}=1500\;\text{m/s}$ [62], and viscosity μ=0.015Pas [2]. Although this scenario simplifies the true complexity of biological media, it provides a valuable framework to understand the sensitivity of the nonlinear parameter β to changes in elastic properties.

As in the previous cases, appropriate source amplitudes are selected. The corresponding values for the biological soft tissue of Table 6, together with the sound speed, are summarized in Table 7.

**Table 6:** Shear modulus G of biological soft tissue media [32].

Medium	Shear modulus G (kPa)
Skin	0.37
Fat	3.3
Muscle	6.7
Breast cancer	31

**Table 7:** Sound speed in the bubbly biological soft tissue medium, $c_{bf}$, at frequency f=20 kHz for bubbles with radius $R_{0}=4.5\;\mu \text{m}$ and density $N_{g}=1\times 10^{11}\;m^{-3}$, and source amplitude $p_{0}$ suitable for the calculation of the nonlinear parameter β for the media listed in Table 6.

Medium	Sound speed $c_{bf}$ (m/s)	Source amplitude $p_{0}$ (Pa)
Skin	1195	2000
Fat	1201	2100
Muscle	1207	2200
Breast cancer	1242	2900

The nonlinear parameter β is computed for each case to assess the influence of shear elasticity on the generation of higher harmonics in bubbly viscoelastic media.

**Fig. 7:**
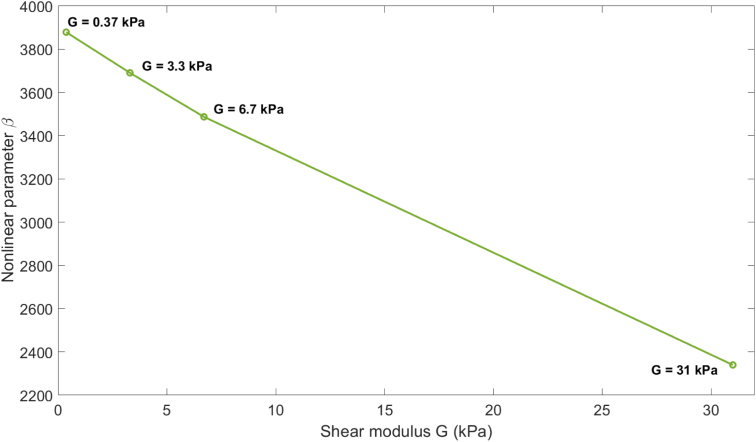
Variation of the nonlinear parameter β with shear modulus for the bubbly biological soft tissues presented in Table 6. $R_{0}=4.5\;\mu \text{m}$, $N_{g}=1\times 10^{11}\;m^{-3}$, f=20kHz.

The results show a clear trend, as can be observed in Fig. 7, that displays the variation of the nonlinear parameter β with the shear modulus for the bubbly biological soft tissues presented in Table 6: the nonlinear parameter decreases as the shear modulus increases. Specifically, the highest β obtained is for skin β=3878, followed by fat β=3690, muscle β=3487.2, and finally breast cancer tissue β=2339. This inverse relationship suggests that stiffer tissues tend to suppress nonlinear wave propagation more effectively. These findings are consistent with the observations from the gelatin and liver media discussed previously, where higher stiffness also leads to reduced second harmonic generation.

Thus, the analysis supports the conclusion that shear elasticity plays a significant role in modulating acoustic nonlinearity in biphasic media, especially in the presence of gas bubbles.

#### 3.2.3 Effects of medium viscosity on the nonlinear parameter

Having established that shear elasticity significantly affects the nonlinear parameter β, we now turn our attention to the role of viscosity as a dissipative property of the medium. Viscosity governs energy losses during bubble oscillations [30], [63] and is therefore expected to influence nonlinear wave propagation and harmonic generation in bubbly media.

We consider a set of bubbly fluids composed of aqueous glycerol solutions [64] with increasing dynamic viscosity, as listed in Table 8. The density in the medium is considered the same as in the previous section, $\rho =1000\;{\text{kg/m}}^{3}$. The sound speed in the bubbly medium here is $c_{bf}=1194.7\;\text{m/s}$.

**Fig. 8:**
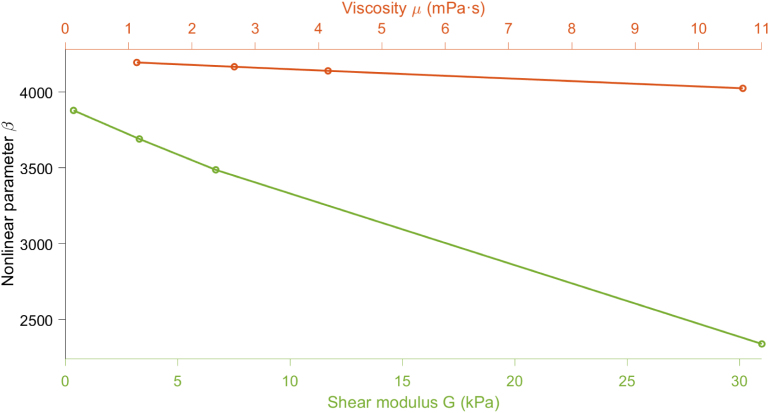
Variation of the nonlinear parameter β for the bubbly viscoelastic media presented in Table 6, Table 8. $R_{0}=4.5\;\mu \text{m}$, $N_{g}=1\times 10^{11}\;m^{-3}$, f=20kHz.

The computed values of β exhibit a moderate decreasing trend with increasing viscosity: β=4183.3 for GLY04, β=4151.3 for GLY35, β=4120.9 for GLY47, and β=3990.8 for GLY60 (see Fig. 8). Although the variation is relatively small, this behavior confirms that viscosity contributes to a reduction in the nonlinear acoustic response, likely due to enhanced damping of bubble oscillations (see Table 9). Fig. 8 compares the influence of viscosity and elasticity on the nonlinear parameter β for the viscoelastic bubbly media defined in Table 6, Table 8. While the red curve confirms the weak influence of viscosity, producing only a minor reduction in β (from 4183.3 to 3990.8) across the studied range, the green curve reveals a much stronger dependence on the shear modulus G. As G increases from 0.37 to 31kPa, the nonlinear parameter β decreases significantly from 3879 to 2340. This trend suggests that the elastic properties of the medium have a far more pronounced effect on the acoustic nonlinearity than the viscous dissipation. Therefore, the stiffness of the host medium plays a dominant role in determining the nonlinear acoustic response in bubbly viscoelastic media.

**Table 8:** Dynamic viscosity μ of the considered aqueous glycerol solutions [64].

Medium	Viscosity μ (mPa s)
GLY04	1.13
GLY35	2.67
GLY47	4.15
GLY60	10.7

**Table 9:** Source amplitude $p_{0}$ suitable for the calculation of the nonlinear parameter β for the media listed in Table 8, at frequency of f=20 kHz for bubbles with radius $R_{0}=4.5\;\mu \text{m}$ and density $N_{g}=1\times 10^{11}\;m^{-3}$.

Medium	Source amplitude $p_{0}$ (Pa)
GLY04	2400
GLY35	2550
GLY47	2650
GLY60	2850

Future work will explore perturbative analytical approaches to approximate the fundamental and second harmonic components of acoustic pressure, providing a complementary tool to the present numerical results.

The agreement of these findings with previous studies reinforces the predictive value of the proposed model for exploring nonlinear ultrasound propagation in such media, particularly in soft biological tissues or engineered materials with embedded bubbles.

## 4 Conclusions

We have developed a numerical framework to study nonlinear ultrasonic wave propagation in bubbly viscoelastic media by coupling the wave equation with a modified Rayleigh–Plesset equation, expressed in terms of bubble volume variation and incorporating the Kelvin–Voigt model for the surrounding medium. This formulation allows for direct analysis of the influence of viscoelastic properties on acoustic nonlinearity under realistic soft tissue conditions. The model has been numerically solved to investigate both the linear and nonlinear regimes of ultrasonic wave propagation across different representative viscoelastic materials and media with increasing shear elasticity. Our results show that the bubbly liquid in the absence of elasticity exhibits the strongest nonlinear distortions. As the shear modulus increases, significantly higher source amplitudes are required to initiate nonlinear behavior, resulting in attenuated harmonic amplitudes. This trend is supported by the definition of laws of the second and third harmonics amplitudes vs. excitation at the source obtained from their polynomial fits, which reflect the nonlinear response of the system under varying excitation levels and viscoelastic properties. We have quantified these nonlinear effects by evaluating the nonlinear parameter β using a finite-amplitude method in bubbly viscoelastic systems. The computed values of β are several orders of magnitude higher than those typical of homogeneous media due to the presence of gas bubbles. Furthermore, by analyzing a set of biphasic viscoelastic tissues with identical bubble concentrations and varying only the shear modulus, we isolated the role of elasticity. The results confirm that shear elasticity significantly reduces nonlinearity in ultrasound propagation and mitigates harmonic generation: β decreases as the shear modulus increases, demonstrating a clear inverse relationship between tissue stiffness and acoustic nonlinearity. Finally, the role of viscosity has been assessed and found to have a weak influence on the nonlinear parameter within the range studied here. This confirms that, under typical soft tissue conditions, elasticity plays a much more significant role than viscous dissipation in the regulation of nonlinear acoustic behavior. These findings support the interpretation that the nonlinear parameter β is not an isolated material property, but rather a reflection of the underlying physical microstructure of the tissue. Our results, consistent with existing literature, support the model as a valuable tool to study nonlinear ultrasound in viscoelastic bubbly media.

## CRediT authorship contribution statement

**Elena V. Carreras-Casanova:** Writing – review & editing, Writing – original draft, Visualization, Validation, Software, Resources, Methodology, Investigation, Formal analysis, Data curation, Conceptualization. **María Teresa Tejedor-Sastre:** Writing – review & editing, Writing – original draft, Software, Investigation, Data curation. **Christian Vanhille:** Writing – review & editing, Writing – original draft, Visualization, Validation, Supervision, Software, Resources, Project administration, Methodology, Investigation, Funding acquisition, Formal analysis, Data curation, Conceptualization.

## Funding

This work was supported by the 10.13039/501100007511Universidad Rey Juan Carlos through the Pre-Doctoral Grant No. C1PREDOC23-023.

## Declaration of competing interest

The authors declare the following financial interests/personal relationships which may be considered as potential competing interests: Elena V. Carreras-Casanova reports financial support was provided by Universidad Rey Juan Carlos. If there are other authors, they declare that they have no known competing financial interests or personal relationships that could have appeared to influence the work reported in this paper.

## References

[1] Lapin N.A., Gill K., Shah B.R., Chopra R. (2020). Consistent opening of the blood brain barrier using focused ultrasound with constant intravenous infusion of microbubble agent. Sci. Rep..

[2] Zilonova E., Solovchuk M., Sheu T. (2018). Bubble dynamics in viscoelastic soft tissue in high-intensity focal ultrasound thermal therapy. Ultrason. Sonochemistry.

[3] Tejedor Sastre M.T., Vanhille C. (2017). A numerical model for the study of the difference frequency generated from nonlinear mixing of standing ultrasonic waves in bubbly liquids. Ultrason. Sonochemistry.

[4] Leighton T. (1994).

[5] Hamilton M., Blackstock D. (1998). https://books.google.es/books?id=k-kWozCE0jIC.

[6] Kuznetsov V.V., Nakoryakov V.E., Pokusaev B.G., Shreiber I.R. (1978). Propagation of perturbations in a gas-liquid mixture. J. Fluid Mech..

[7] Drumheller D.S., Kipp M.E., Bedford A. (1982). Transient wave propagation in bubbly liquids. J. Fluid Mech..

[8] Kanagawa T., Yano T., Watanabe M., Fujikawa S. (2011). Nonlinear wave equation for ultrasound beam in nonuniform bubbly liquids. J. Fluid Sci. Technol..

[9] Kanagawa T. (2015). Two types of nonlinear wave equations for diffractive beams in bubbly liquids with nonuniform bubble number density. J. Acoust. Soc. Am..

[10] Kagami S., Kanagawa T. (2022). Weakly nonlinear propagation of focused ultrasound in bubbly liquids with a thermal effect: Derivation of two cases of Khokolov–Zabolotskaya–Kuznetsoz equations. Ultrason. Sonochemistry.

[11] Vanhille C., Campos-Pozuelo C. (2009). Numerical simulation of nonlinear ultrasonic standing waves in bubbly liquid. Int. J. Nonlinear Sci. Numer. Simul..

[12] Vanhille C., Campos-Pozuelo C. (2011). Nonlinear ultrasonic standing waves: Two-dimensional simulations in bubbly liquids. Ultrason. Sonochemistry.

[13] Vanhille C., Campos-Pozuelo C. (2013). Numerical simulations of three-dimensional nonlinear acoustic waves in bubbly liquids. Ultrason. Sonochemistry.

[14] Dollet B., Marmottant P., Garbin V. (2019). Bubble dynamics in soft and biological matter. Annu. Rev. Fluid Mech..

[15] Yang X., Church C.C. (2005). A model for the dynamics of gas bubbles in soft tissue. J. Acoust. Soc. Am..

[16] Gaudron R., Warnez M.T., Johnsen E. (2015). Bubble dynamics in a viscoelastic medium with nonlinear elasticity. J. Fluid Mech..

[17] Warnez M.T., Johnsen E. (2015). Numerical modeling of bubble dynamics in viscoelastic media with relaxation. Phys. Fluids.

[18] Jamburidze A., De Corato M., Huerre A., Pommella A., Garbin V. (2017). High-frequency linear rheology of hydrogels probed by ultrasound-driven microbubble dynamics. Soft Matter.

[19] Kagami S., Kanagawa T. (2023). Weakly nonlinear focused ultrasound in viscoelastic media containing multiple bubbles. Ultrason. Sonochemistry.

[20] Murakami K., Yamakawa Y., Zhao J., Johnsen E., Ando K. (2021). Ultrasound-induced nonlinear oscillations of a spherical bubble in a gelatin gel. J. Fluid Mech..

[21] Wang Y., Chen D., Wu P. (2023). Multi-bubble scattering acoustic fields in viscoelastic tissues under dual-frequency ultrasound. Ultrason. Sonochemistry.

[22] Campbell I.J., Pitcher A.S. (1958). Shock waves in a liquid containing gas bubbles. Proc. R. Soc. Lond. Ser. A Math. Phys. Sci..

[23] Fogler H., Goddard J. (1970). Collapse of spherical cavities in viscoelastic fluids. Phys. Fluids.

[24] Tanasawa I., Yang W.-J. (1970). Dynamic behavior of a gas bubble in viscoelastic liquids. J. Appl. Phys..

[25] Allen J.S., Roy R.A. (2000). Dynamics of gas bubbles in viscoelastic fluids. I. Linear viscoelasticity. J. Acoust. Soc. Am..

[26] Hua C., Johnsen E. (2013). Nonlinear oscillations following the Rayleigh collapse of a gas bubble in a linear viscoelastic (tissue-like) medium. Phys. Fluids.

[27] Doinikov A.A., Marmottant P. (2018). Natural oscillations of a gas bubble in a liquid-filled cavity located in a viscoelastic medium. J. Sound Vib..

[28] Vlaisavljevich E., Maxwell A., Warnez M., Johnsen E., Cain C.A., Xu Z. (2014). Histotripsy-induced cavitation cloud initiation thresholds in tissues of different mechanical properties. IEEE Trans. Ultrason. Ferroelectr. Freq. Control.

[29] Maxwell A.D., Cain C.A., Hall T.L., Fowlkes J.B., Xu Z. (2013). Probability of cavitation for single ultrasound pulses applied to tissues and tissue-mimicking materials. Ultrasound Med. Biol..

[30] Carreras-Casanova E.V., Vanhille C. (2025).

[31] Qin D., Zou Q., Lei S., Wang W., Li Z. (2021). Nonlinear dynamics and acoustic emissions of interacting cavitation bubbles in viscoelastic tissues. Ultrason. Sonochemistry.

[32] Hasegawa T., Kanagawa T. (2023). Effect of liquid elasticity on nonlinear pressure waves in a viscoelastic bubbly liquid. Phys. Fluids.

[33] Naugolnykh K., Ostrovsky L. (1998).

[34] Vanhille C., Pantea C., Sinha D.N. (2017). Acoustic characterization of fluorinert FC-43 liquid with helium gas bubbles: Numerical experiments. Shock. Vib..

[35] Hemmi K., Kanagawa T. (2025). Impact of a bubble–bubble interaction on nonlinear acoustic properties of pressure waves in a non-dilute bubbly liquid. Results Eng..

[36] Beyer R.T. (1960). Parameter of nonlinearity in fluids. J. Acoust. Soc. Am..

[37] Law W., Frizzell L., Dunn F. (1985). Determination of the nonlinearity parameter B/A of biological media. Ultrasound Med. Biol..

[38] Fox F.E., Wallace W.A. (1954). Absorption of finite amplitude sound waves. J. Acoust. Soc. Am..

[39] Law W.K., Frizzell L.A., Dunn F. (1983). Comparison of thermodynamic and finite amplitude methods of B/A measurement in biological materials. J. Acoust. Soc. Am..

[40] Chien L.D., Cormack J.M., Everbach E.C., Hamilton M.F. (2022). Determination of nonlinearity parameter B/A of liquids by comparison with solutions of the three-dimensional Westervelt equation. Proc. Meet. Acoust..

[41] Varray F., Basset O., Tortoli P., Cachard C. (2011). Extensions of nonlinear B/A parameter imaging methods for echo mode. IEEE Trans. Ultrason. Ferroelectr. Freq. Control.

[42] Mast T.D. (2000). Empirical relationships between acoustic parameters in human soft tissues. Acoust. Res. Lett. Online.

[43] Panfilova A., van Sloun R.J.G., Wijkstra H., Sapozhnikov O.A., Mischi M. (2021). A review on B/A measurement methods with a clinical perspective. J. Acoust. Soc. Am..

[44] Zhang D., Gong X.-F. (1999). Experimental investigation of the acoustic nonlinearity parameter tomography for excised pathological biological tissues. Ultrasound Med. Biol..

[45] Zhang D., fen Gong X., Chen X. (2001). Experimental imaging of the acoustic nonlinearity parameter B/A for biological tissues via a parametric array. Ultrasound Med. Biol..

[46] Zhang J., Kuhlenschmidt M.S., Dunn F. (1991). Influences of structural factors of biological media on the acoustic nonlinearity parameter B/A. J. Acoust. Soc. Am..

[47] Panfilova A., Chen X., Widdershoven C., Freund J.E., Heijink D.S., Zondervan P., van Sloun R.J., Sapozhnikov O.A., Wijkstra H., Mischi M. (2022). B/a measurement of clear cell renal cell carcinoma versus healthy kidney tissue. Ultrasound Med. Biol..

[48] Dunn F., Law W., Frizzell L. (1982). Nonlinear ultrasonic propagation in biological media. Br. J. Cancer. Suppl..

[49] Chen P., Pollet A.M., Panfilova A., Zhou M., Turco S., den Toonder J.M., Mischi M. (2022). Acoustic characterization of tissue-mimicking materials for ultrasound perfusion imaging research. Ultrasound Med. Biol..

[50] Duck F.A. (2002). Nonlinear acoustics in diagnostic ultrasound. Ultrasound Med. Biol..

[51] Heyns J.D., Ahmed Mohamed E.T., Declercq N.F. (2021). Non-linear ultrasonic and viscoelastic properties of gelatine investigated in the temperature range of 30°C–60°C. Phys. Fluids.

[52] Jafarzadeh E., Amini M.H., Sinclair A.N. (2021). Determination of the ultrasonic non-linearity parameter B/A versus frequency for water. Ultrasound Med. Biol..

[53] Hamilton M.F. (2025). The nonlinearity parameter B/A. J. Acoust. Soc. Am..

[54] Everbach E.C., Zhu Z., Jiang P., Chu B.T., Apfel R.E. (1991). A corrected mixture law for B/A. J. Acoust. Soc. Am..

[55] Bjørno L. (1982). Acoustic nonlinearity of bubbly liquids. Appl. Sci. Res..

[56] Zabolotskaya E.A., Soluyan S.I. (1973). https://api.semanticscholar.org/CorpusID:203035941.

[57] Vanhille C. (2021). A fourth-order approximation Rayleigh-Plesset equation written in volume variation for an adiabatic-gas bubble in an ultrasonic field: Derivation and numerical solution. Results Phys..

[58] Vanhille C., Lavie A., Campos-Pozuelo C. (2007).

[59] Smith G.D. (1985).

[60] Versteeg H.K. (2007).

[61] Pantea C., Osterhoudt C.F., Sinha D.N. (2013). Determination of acoustical nonlinear parameter β of water using the finite amplitude method. Ultrasonics.

[62] Wells P., Liang H. (2011). Medical ultrasound: Imaging of soft tissue strain and elasticity. J. R. Soc. Interface / R. Soc..

[63] Filonets T., Solovchuk M. (2022). GPU-accelerated study of the inertial cavitation threshold in viscoelastic soft tissue using a dual-frequency driving signal. Ultrason. Sonochemistry.

[64] Crha J., Orvalho S., Ruzicka M.C., Shirokov V., Jerhotová K., Pokorny P., Basařová P. (2024). Bubble formation and swarm dynamics: Effect of increased viscosity. Chem. Eng. Sci..

